# Effect of Artificial Saliva Modification on Corrosion Resistance of Metal Oxide Coatings on Co-Cr-Mo Dental Alloy

**DOI:** 10.3390/ma17215166

**Published:** 2024-10-23

**Authors:** Bożena Łosiewicz, Patrycja Osak, Karolina Górka-Kulikowska, Joanna Maszybrocka

**Affiliations:** 1 Institute of Materials Engineering, Faculty of Science and Technology, University of Silesia in Katowice, 75 Pułku Piechoty 1A, 41-500 Chorzów, Poland; 2 Department of Biomaterials and Experimental Dentistry, Poznan University of Medical Sciences, 60-812 Poznań, Poland

**Keywords:** Co-Cr-Mo dental alloy, corrosion resistance, pitting corrosion, saliva, TiO_2_-ZrO_2_ sol–gel coating

## Abstract

Surface modifications not only improve the corrosion resistance of Co-Cr-Mo dental alloys (Bego Wirobond^®^ C) but also ensure their long-term performance and reliability in dental applications. This paper describes the preparation of single-layer TiO_2_-ZrO_2_ sol–gel coatings on the Co-Cr-Mo dental alloy using the method of dip-coating. The TiO_2_-ZrO_2_ sol–gel coatings were sintered at 300 and 500 °C. SEM analysis shows that sintering at 300 °C produces a uniform, slightly dense structure without micro-cracks, while sintering at 500 °C results in a denser structure with micro-cracks due to higher stress and shrinkage. EDS confirms that sintering temperature affects the elemental composition of the coating, with higher temperatures causing the volatilization or diffusion of Ti and Zr. Roughness measurements indicate that the Ra value increases with the sintering temperature, meeting dental application requirements. Electrochemical measurements by open-circuit potential, EIS, and cyclic potentiodynamic curves demonstrate that sintering temperature and saliva composition affect corrosion resistance, with NaF and mouthwashes (Listerine Total Care Teeth Protection^®^ and Meridol^®^) generally increasing charge transfer resistance and double-layer capacitance. The ceramic TiO_2_-ZrO_2_ coatings significantly reduce pitting corrosion susceptibility at physiological and acidic pH, with the 500 °C sintered coating showing better protective properties. These findings highlight the potential of TiO_2_-ZrO_2_ coatings in enhancing the performance of Co-Cr-Mo dental alloys.

## 1. Introduction

In modern dentistry, a variety of biomaterials are required to address different dental needs, including restorations, prosthetics, and preventive treatments [1,2,3]. Base metal alloys are often used in dentistry in many denture infrastructure and tools [4,5,6,7,8,9]. Co-Cr-Mo alloys belonging to this group are known for their high strength and hardness, which make them suitable for applications requiring durability and resistance to wear [8,10,11]. These alloys exhibit excellent biocompatibility, which is crucial for use in dental and orthopedic applications where they come into direct contact with living tissues [12,13]. Co-Cr-Mo alloys have a high melting point, which is beneficial for their processing and fabrication into various dental and orthopedic components [10]. They have good thermal conductivity, which can be advantageous in certain applications where heat management is important [14]. Co-Cr-Mo alloys exhibit exceptional long-term corrosion resistance in environments with corrosive substances due to their chemical composition and the protective oxide layer formed by chromium [12,13,15]. However, specific conditions such as mechanical stress and exposure to certain ions can influence their long-term performance. These alloys are non-magnetic, ensuring they do not interfere with medical imaging equipment, which makes them suitable for medical implants [16]. They also offer ductility and toughness, which are important for their use in dental prosthetics and other medical devices [17,18]. Co-Cr-Mo alloys are also well suited for various manufacturing processes, including casting and machining, which allows for the precise customization of dental appliances [19,20].

The corrosion resistance of Co-Cr-Mo dental alloys is a critical factor in their use, particularly in dental applications where they are exposed to various corrosive environments [8,12,13,15,21,22,23,24,25,26,27,28,29,30]. The alloy exhibits several characteristics that enhance its corrosion resistance. This alloy contains significant amounts of Cr and Mo, which are known for their corrosion-resistant properties. The presence of these elements helps in forming a passive oxide layer on the surface of the alloy, which protects it from further corrosion [8,13,26,27]. The microstructure of Co-Cr-Mo alloys, including the distribution of carbides and the dendritic structure of the solid solution, can influence their corrosion resistance. The proper control of these microstructural features can enhance the alloy’s resistance to corrosion [21]. The method of manufacturing can also affect the corrosion resistance of Co-Cr-Mo alloys [12,21,22,23,24,25,28]. Studies have shown that Co-Cr-Mo alloys exhibit capacitive behavior in electrochemical impedance spectroscopy (EIS) tests, indicating a high resistance to corrosion [8,26,27]. The surface roughness of Co-Cr-Mo alloys can influence their corrosion resistance. A smoother surface reduces the contact area with corrosive substances, thereby enhancing the alloy’s resistance to corrosion [21,28]. These characteristics collectively contribute to the high corrosion resistance of Co-Cr-Mo dental alloys, making them a preferred material for use in dental and orthopedic applications.

However, the corrosion of Co-Cr-Mo dental alloys during long-term use can be a significant concern due to the potential release of metal ions and corrosion products that may lead to local tissue reactions or systemic effects [15]. The corrosion resistance of the alloy is influenced by several factors, including the alloy composition, surface finish, presence of crevices, and galvanic coupling with other metals [24,26,28]. The results of studies also suggest that modifications of the saliva chemical composition by the addition of fluoride, the pH change, the presence of biofilms, and the use of specific mouthwashes can significantly influence the corrosion resistance of Co-Cr-Mo dental alloys [8,29,30].

Surface modifications of Co-Cr-Mo dental alloys by surface amorphization [31], laser surface modification [32], and surface polishing used to reduce surface roughness [8] can also improve the corrosion resistance of Co-Cr-Mo dental alloys, ensuring their long-term performance and reliability in dental applications.

The sol–gel method is particularly interesting because it offers a controlled and versatile approach for the synthesis of metal oxide coatings on dental alloys, providing enhanced corrosion resistance and improved surface properties [33,34,35,36,37]. This method involves the conversion of a metal alkoxide precursor into a sol, which is a colloidal solution, and then into a gel, which is a solidified form of the sol. The gel is a three-dimensional network of interconnected particles that can be solidified by drying or heat treatment. It can be applied to the metallic surface by techniques such as dipping, spraying, or spin-coating [33,35,36,37]. The gel adheres to the substrate and can be cured to form a protective solid oxide coating [35,36,37]. Sol–gel coatings can be modified in many ways to achieve excellent biocompatibility and corrosion resistance, making them an attractive solution in orthodontics and medicine [35,36]. In the sol–gel coating production process, the material passes from the liquid phase (sol) to the solid phase (gel) through the controlled polymerization of particles present in the solution. The result is a thin, uniform coating that can be applied to various surfaces, such as metals used in orthodontic appliances [33,35,36].

To enhance biocompatibility, oxide precursors in the form of various inorganic metal compounds are added to sol–gel coatings [38,39,40]. Silicates (Ormosils) are precursors for sol–gel-generated SiO_2_-based coatings, which are frequently studied for their potential in biocompatible applications [38]. Titania (TiO_2_) is used to modify titanium surfaces, enhancing biocompatibility [39]. Zirconia (ZrO_2_) is also used to modify titanium surfaces, contributing to biocompatibility [39]. Hybrid Organic–Inorganic (OIH) precursors are a combination of organic and inorganic components, synthesized using sol–gel methods to enhance biocompatibility and corrosion resistance [38,40]. Phosphorus-containing compounds are used to modify sol–gel coatings, aiming to improve both biocompatibility and corrosion protection behavior [40]. These precursors are chosen based on their ability to form stable oxide networks and their compatibility with biological systems, making them suitable for applications requiring enhanced biocompatibility. Recently, TiO_2_-ZrO_2_ sol–gel coatings were developed to enhance the corrosion resistance of stainless steel for applications in medicine [35,36].

In this work, we continue our interest in the in vitro corrosion resistance of Co-Cr-Mo dental alloys. In a previous study [8], we reported on the results of investigations on the corrosion behavior of the Co-Cr-Mo dental alloy in various artificial saliva environments and a physiological saline solution. The environments were modified with NaF and mouthwashes containing alcoholic (Meridol^®^ by Colgate-Palmolive Company, New York, NY, USA) and aqueous (Listerine Total Care Teeth Protection^®^ by McNeil Consumer Healthcare McNeil-PPC, Inc., Fort Washington, PA, USA) solvents to simulate different oral conditions. It was proven that the corrosion resistance of the Co-Cr-Mo dental alloy can be significantly affected by the composition of the oral environment, particularly with the addition of fluoride ions, which can enhance the protective passive layer on the alloy surface. The aim of this work is to produce for the first time TiO_2_-ZrO_2_ sol–gel coatings sintered at 300 and 500 °C on the commercial Co-Cr-Mo dental alloy Bego Wirobond^®^ C by BEGO Bremer Goldschlägerei Wilh. Herbst GmbH & Co. KG, Bremen, Germany in order to increase its pitting corrosion resistance in the artificial saliva environment. This study provides new insights into the performance of ceramic TiO_2_-ZrO_2_ coatings on Co-Cr-Mo substrates in simulated oral environments, which is critical for ensuring the safety, effectiveness, and longevity of dental treatments and devices.

The choice of TiO_2_-ZrO_2_ sol–gel coatings on Co-Cr-Mo dental alloys is supported by several key factors that enhance their functionality and performance, making them an ideal choice for modern dental implant applications [35,41,42,43,44,45,46,47,48]. TiO_2_-ZrO_2_ coatings can improve the mechanical properties of Co-Cr-Mo alloys. ZrO_2_ is known for its high hardness and toughness, which can enhance the wear resistance and overall durability of dental implants [41]. TiO_2_, when doped with certain elements like silver and gallium, exhibits antibacterial properties. This is particularly beneficial in dental applications where preventing bacterial adhesion and biofilm formation is crucial for maintaining oral health and preventing infections [42]. TiO_2_-ZrO_2_ coatings can be formulated to have high transparency, which is important for aesthetic dental restorations. This property allows the natural color of the underlying tooth structure to show through, enhancing the cosmetic appeal of dental implants [41]. The sol–gel technique used to apply these coatings ensures a strong adhesion between the coating and the substrate, which is essential for maintaining the integrity and functionality of dental implants over time. Electrochemical pretreatment methods can further enhance the adhesion strength of these coatings on Co-Cr-Mo substrates [43]. TiO_2_-ZrO_2_ coatings are known for their high biocompatibility and can improve the biological performance of dental implants. These coatings can enhance the adhesion of bone to the implant surface, which is crucial for long-term stability and integration of dental implants [41]. ZrO_2_ is known for its excellent corrosion resistance, which is particularly important in dental applications where the alloy is exposed to various oral fluids. The addition of TiO_2_ further enhances this property, making the coating more durable and resistant to degradation in the oral environment [35]. Corrosion resistance in artificial saliva is vital for long-term dental applications as it affects both biocompatibility and mechanical integrity. It is essential for preventing adverse reactions and ensuring the longevity of dental treatments [29,44]. Understanding corrosion characteristics in artificial saliva helps in selecting materials that are less likely to corrode and more suitable for long-term clinical use [45,46]. Modifying artificial saliva with substances like fluoride can enhance corrosion resistance, supporting oral health and reducing corrosion-related issues [8,47]. This knowledge is crucial for selecting non-precious metal alloys that perform well in the complex and dynamic oral environment and for developing maintenance strategies that enhance clinical outcomes and patient well-being [48].

## 2. Materials and Methods

### 2.1. Preparation of Material Surface

The test material is the dental Co-Cr-Mo alloy Bego Wirobond^®^ C (BEGO Bremer Goldschlägerei Wilh. Herbst GmbH & Co. KG, Bremen, Germany), meeting the requirements of the standards ISO 22674 [49] and ISO 9693-1 [50]. The Co-Cr-Mo alloy is an approved class IIa medical device, which is manufactured in accordance with the ISO 13485 standard [51]. Bego Wirobond^®^ C by BEGO Bremer Goldschlägerei Wilh. Herbst GmbH & Co. KG, Bremen, Germany containing Co 59.8(8) wt.%, Cr 31.5(4) wt.%, and Mo 8.8(6) wt.%. is used in dental practice for the production of partial dentures, clasps, veneered crowns, long-span bridgework or bridges with small cross-sections, bars, retainers, and implant-supported superstructures [8].

Co-Cr-Mo Bego Wirobond^®^ C by BEGO Bremer Goldschlägerei Wilh. Herbst GmbH & Co. KG, Bremen, Germany alloy samples were cut into 5 mm thick disks from 15 mm thick cylinders. The samples were ground on #320- to #2500-grit sandpaper and then polished using a colloidal SiO_2_ suspension (Buehler Ltd., Lake Bluff, IL, USA). In the next step, the samples were degreased in acetone (Avantor Performance Materials Poland SA, Gliwice, Poland) and then in ultrapure water (Milli-Q Advantage A10 Water Purification System, Millipore SAS, Molsheim, France) in an ultrasonic cleaner for 20 min (VWR International, Radnor, PA, USA).

### 2.2. Production of TiO_2_-ZrO_2_ Sol–Gel Coatings

**Figure 1:**
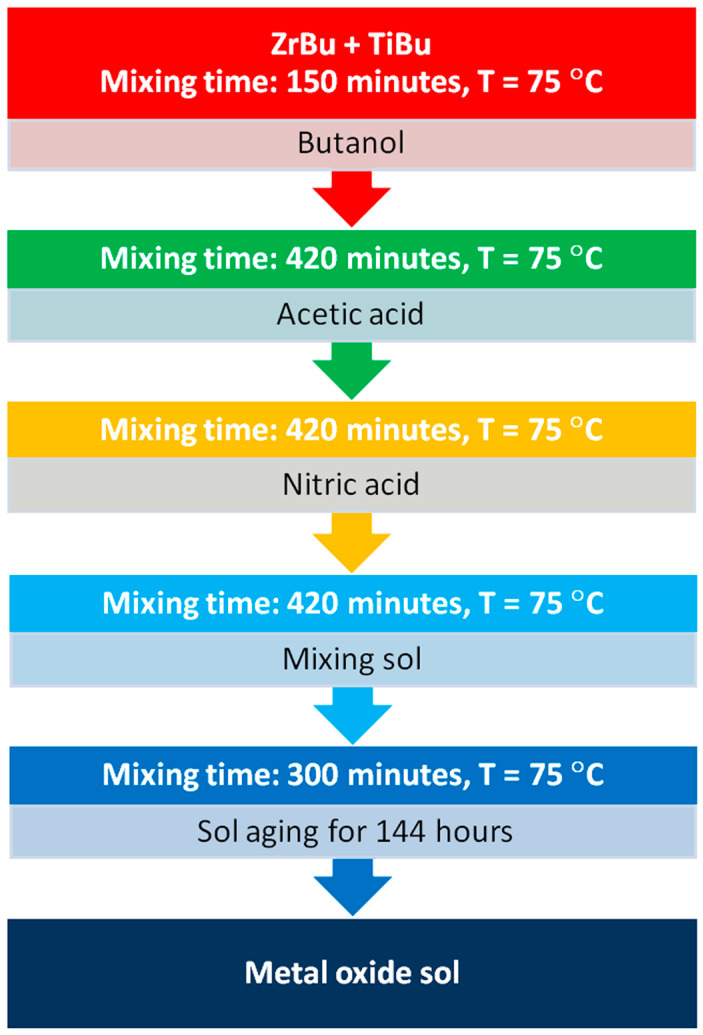
Flowchart of sol–gel process for dip-coating technique.

The surface of the Co-Cr-Mo Bego Wirobond^®^ C by BEGO Bremer Goldschlägerei Wilh. Herbst GmbH & Co. KG, Bremen, Germany alloy was modified by depositing metal oxide coatings using the sol–gel method. To visualize the sol–gel process for the dip-coating technique, a flowchart with five steps, including arrows to show the progression from one step to the next, is presented in Figure 1. Each step is detailed with specific conditions or processes. The single-layer TiO_2_-ZrO_2_ sol–gel coatings were deposited by immersing the samples in a colloidal solution consisting of two precursors—zirconium (1 M ZrBu) and titanium (1 M TiBu). The samples were immersed in the solution prepared in this way at a temperature of 75 °C using the following procedure: immersion for 150 min, rinsing the samples with butanol—CH_3_(CH_2_)_3_OH (Sigma-Aldrich, Saint Louis, MI, USA)—immersion in precursors for 420 min and rinsing in acetic acid CH_3_COOH (Sigma-Aldrich, Saint Louis, MI, USA), immersion for 420 min, then rinsing with nitric acid (Avantor Performance Materials Poland SA, Gliwice, Poland), immersion for 420 min and the formation of a sol layer, immersion for 300 min, and then the aging of the sol for 144 h in air. In the next stage, the TiO_2_-ZrO_2_ sol–gel coatings obtained in this way were sintered at 300 °C and 500 °C for 1 h in an argon atmosphere.

The choice of sintering temperatures of 300 and 500 °C for the TiO_2_-ZrO_2_ sol–gel coating is justified by several factors related to the material’s phase stability and corrosion resistance [35,36,52,53,54]. According to the literature, TiO_2_-ZrO_2_ sol–gel coatings remain in the amorphous phase up to 500 °C. This temperature is crucial as it marks the onset of crystallization, which is essential for the development of the desired crystalline structure of the coating. This phase transformation is critical for the material’s properties, such as mechanical strength and chemical stability [35]. At temperatures above 500 °C, the presence of oxygen vacancies and lattice distortion in ZrO_2_ is confirmed [52]. These defects can influence the electrical properties of the coating. Sintering at 300 and 500 °C ensures that the coating remains in the amorphous phase, which is beneficial for applications requiring specific electrical properties, as amorphous materials do not exhibit long-range order and can have unique properties not found in crystalline materials. Sintering at 500 °C enhances the corrosion resistance of the TiO_2_-ZrO_2_ coating. This temperature is effective in stabilizing the coating’s structure, which in turn improves its resistance to corrosive environments [36]. Annealing at 500 °C is a balance that ensures the structural integrity of the coating without causing excessive thermal damage. It is a temperature that is high enough to induce necessary phase changes and low enough to avoid the formation of unwanted phases or the decomposition of the coating components [53,54]. Additionally, it was found that sol–gel TiO_2_-ZrO_2_ coatings with an amorphous structure and calcined at 400 °C significantly enhance the corrosion properties of AISI 316 L stainless steel [36]. Although 400 and 500 °C are slightly higher than 300 °C, this indicates that temperatures in this range are effective for improving corrosion resistance, suggesting that 300 °C could also be beneficial for similar applications. Lower sintering temperatures like 300 °C are generally more energy-efficient and safer, as they require less energy input and pose fewer risks of thermal decomposition or phase transformations that could degrade the coating’s properties.

The dip-coating technique is a method used to deposit a thin film on a substrate by immersing the substrate into a coating solution and then withdrawing it at a controlled rate. This process involves five main steps: immersion, startup, deposition, drainage, and evaporation [55]. Dip-coating is a relatively inexpensive and straightforward process compared to other coating methods. It does not require complex equipment, making it accessible for various applications, including dental alloys [56]. The thickness of the deposited film can be precisely controlled by adjusting the withdrawal speed of the substrate from the coating solution. This control is crucial for applications where uniformity is essential, such as in dental alloys where consistent coating is necessary for performance and longevity [55,57]. Dip-coating can be applied to a wide range of substrates, including dental alloys, which may have complex shapes or sizes. This versatility makes it a suitable method for coating dental implants and other dental devices [57]. It can be combined with other techniques like sol–gel to enhance the properties of the coating. This combination can improve the adhesion, durability, and functionality of the coating on dental alloys [55]. While spin-coating is effective for depositing uniform thin films on flat substrates, it requires precise control over the spinning speed and the amount of coating solution. It may not be as effective for irregularly shaped dental alloys [58]. Sputtering is a high-vacuum process that can also deposit thin films with high purity and good adhesion. However, it is more expensive and requires specialized equipment, which may not be feasible for all dental applications [59].

### 2.3. Material Characterization

The microstructure of the TiO_2_-ZrO_2_ sol–gel coatings on the surface of the Co-Cr-Mo substrate was studied using a JEOL JSM-6480 (Peabody, MA, USA) scanning electron microscope (SEM), with a resolution of 3 nm and an accelerating voltage of 20 kV. The local chemical composition of the samples was analyzed using the Energy-Dispersive X-ray Spectroscopy (EDS) technique.

The porosity of TiO_2_-ZrO_2_ sol–gel coatings on a Co-Cr-Mo substrate, after being sintered at temperatures of 300 and 500 °C, was assessed using a computerized porosity testing method. SEM images, which depicted the surface morphology of the sintered coatings, were analyzed using the ImageJ software v1.54k in conjunction with the JPOR macro [60]. This macro is specifically designed to function with ImageJ and utilizes a thresholding technique to quantify porosity from the microscopic images obtained. To calculate porosity using ImageJ, Equation (1) was used:

Porosity (%) = (Total Area of Pores/Total Image Area) × 100.(1)

The surface roughness of the obtained coatings and the Co-Cr-Mo substrate was determined by contact profilometry using a Mitutoyo Surftest SJ-210 profilometer (Mitutoyo Corporation, Kanagawa, Japan). The measurement was performed in accordance with the ISO 21920-3:2022-06 standard [61]. During the measurement, the speed of the measuring needle was 0.5 mm s^−1^, and 4 mm long sections were collected. Five measurements were recorded for each sample, and the result presented in the paper is the average of the measurements.

### 2.4. Corrosion Resistance of TiO_2_-ZrO_2_ Sol–Gel Coatings

In vitro tests of the pitting corrosion resistance of the Co-Cr-Mo alloy were conducted in an artificial saliva solution prepared in accordance with the AFNOR/NF S90-701 standard [62]. First, 0.1M NaF was added to the artificial saliva solutions with pH = 7.4(1) and pH = 5.5(1) to simulate the active ingredient of toothpastes. A total of 15 mL of commercial antiseptic mouthwashes Listerine Total Care Teeth Protection^®^ (McNeil Consumer Healthcare McNeil-PPC, Inc., Fort Washington, PA, USA), which is an alcohol-based mouthwash (21.6% *v*/*v*), and Meridol^®^ (Colgate-Palmolive Company, New York, NY, USA), which is alcohol-free, was added to the artificial saliva solution with pH 7.4(1). Six types of artificial saliva solution were used in the electrochemical measurements, designated as follows: (1) saliva pH = 7.4, (2) saliva pH = 5.5, (3) saliva pH = 7.4 + 0.1M NaF, (4) saliva pH = 5.5 + 0.1M NaF, (5) saliva pH = 7.4 + 15 mL Listerine Total Care Teeth Protection^®^ by McNeil Consumer Healthcare McNeil-PPC, Inc., Fort Washington, PA, USA, and (6) saliva pH = 7.4 + 15 mL Meridol^®^ by Colgate-Palmolive Company, New York, NY, USA.

A three-electrode system was used for this study, where the working electrode (WE) was a Co-Cr-Mo alloy, the counter electrode (CE) was a Pt foil, and the reference electrode (RE) was a saturated calomel electrode (SCE) using the Autolab/PGSTAT12 (Metrohm Autolab B.V., Utrecht, The Netherlands). Electrochemical measurements were performed at 37(2) °C in solutions degassed with argon. The studies were carried out using classical methods such as the open-circuit potential method, in which the potential was recorded for a time (t) of 6000 s, and using cyclic anodic polarization curves, which were recorded in the potential range from 0.15 V more negative than the open-circuit potential (E_OC_) value to 0.2 mV at a polarization rate of 1 mV s^−1^. The studies using the EIS method were performed by recording the EIS spectrum at the E_OC_ potential in the frequency range (f) of 20 kHz–10 mHz with a sinusoidal signal amplitude of 10 mV. EIS data were analyzed using the EQUIVCRT program included in Frequency Response Analyser (FRA) for Windows software v4.9 [63].

## 3. Results and Discussion

### 3.1. SEM Observations of TiO_2_-ZrO_2_ Sol–Gel Coatings

**Figure 2:**
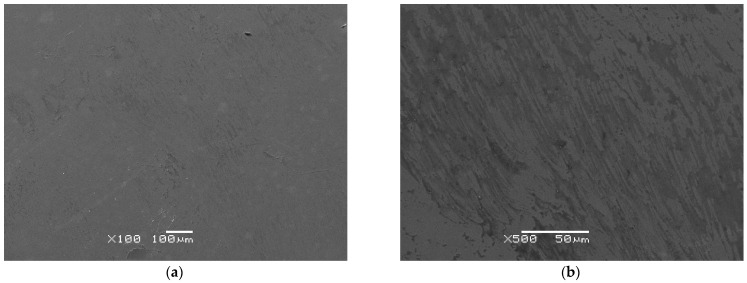

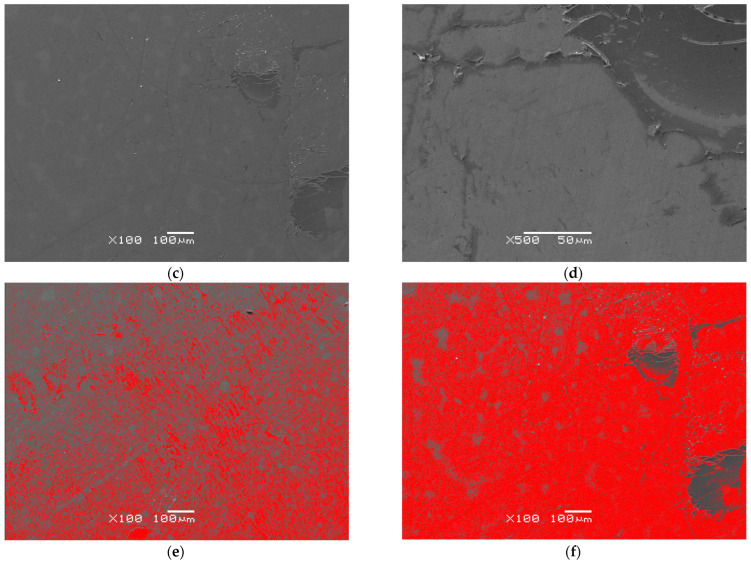
SEM images of the single TiO_2_-ZrO_2_ sol–gel coating deposited on the surface of a Co-Cr-Mo dental alloy after sintering at the following temperatures: (**a**,**b**) 300 °C; (**c**,**d**) 500 °C; (**e**) 300 °C after a computer analysis of porosity; and (**f**) 500 °C after a computer analysis of porosity. Red indicates the color of the applied filter in the porosity measurement using ImageJ software.

Based on SEM images, the surface morphology of TiO_2_-ZrO_2_ sol–gel coatings deposited by the dip-coating method under the proposed conditions on the Co-Cr-Mo alloy surface after sintering at different temperatures was presented. Figure 2a,b show SEM images of the surface morphology of a single-layer TiO_2_-ZrO_2_ sol–gel coating sintered at 300 °C, while Figure 2c,d present SEM images of the surface morphology obtained for the single-layer TiO_2_-ZrO_2_ sol–gel coating over the Co-Cr-Mo substrate after sintering at 500 °C. Comparative SEM images for the polished surface of the Co-Cr-Mo alloy were presented in an earlier work with the EDS analysis results [8].

One can see that sintering influences the surface morphology of TiO_2_-ZrO_2_ sol–gel coatings. SEM images for the single-layer TiO_2_-ZrO_2_ sol–gel coating sintered at 300 °C show a uniform distribution of the obtained ceramic coating on the Co-Cr-Mo alloy substrate (Figure 2a,b). This coating is homogeneous with a slightly dense structure and does not flake off or have visible micro-cracks on the surface. A tight connection of the coating with the Co-Cr-Mo alloy surface is visible. The thickness of the coating was close to 2 μm. SEM images for the single-layer TiO_2_-ZrO_2_ sol–gel coating sintered at 500 °C also show a complete coverage of the alloy surface with the obtained homogeneous ceramic coating characterized by a more dense structure (Figure 2c,d). However, micro-cracks related to the higher sintering temperature are observed on the surface of this coating with a thickness of about 1 μm. Despite the small micro-cracks formed, the coating does not flake or peel off from the alloy surface and is without any deep cracks. The fact that the TiO_2_-ZrO_2_ sol–gel coatings crack during sintering primarily is due to two main reasons: stress induced by film shrinkage and the mismatch of thermal expansion [64]. During the sintering process, the sol–gel coatings undergo significant shrinkage, which can induce internal stresses. These stresses can lead to the formation of cracks as the sol–gel coating contracts and the stress exceeds the material’s tensile strength. The thermal expansion coefficients of the substrate and the coating may not match, leading to differential thermal expansion during the sintering process. This mismatch can also contribute to the development of internal stresses within the coating, potentially causing it to crack due to the internal pressure mismatch between the thermal expansion coefficients of the Co-Cr-Mo substrate and the TiO_2_-ZrO_2_ sol–gel coating. The mean coefficient of thermal expansion at 20–500 °C for the Co-Cr-Mo alloy is 14.1 × 10^−6^/°C [65]. The presence of micro-cracks in the TiO_2_-ZrO_2_ sol–gel coating sintered at 500 °C could affect long-term mechanical properties and potentially lead to failure under dental application conditions. Micro-cracks can significantly reduce the structural integrity and durability of the coating, making it more susceptible to failure under mechanical stress or other environmental factors commonly encountered in dental applications [66,67,68]. It was found that UV irradiation on the sol–gel film could effectively eliminate the micro-cracks in all of the TiO_2_, ZrO_2_, and TiO_2_-ZrO_2_ sol–gel layers, indicating that micro-cracks can compromise the structural integrity of the coatings [66]. Bu et al. [67] reported on the characterization of ZrO_2_–TiO_2_ composite coatings on stainless steel obtained using a dip-coating method. It was proven that the presence of micro-cracks can affect the overall performance and durability of the coating [67]. The multifunctional applications of sol–gel-derived TiO_2_-ZrO_2_ composite coatings were highlighted, noting the importance of minimizing defects like micro-cracks to ensure optimal performance and longevity [68].

Figure 2e,f include SEM images subjected to a computer analysis of porosity which illustrate the TiO_2_-ZrO_2_ sol–gel coatings applied to the Co-Cr-Mo dental alloy after sintering at different temperatures of 300 and 500 °C, respectively. The analysis reveals that the porosity of the coatings is 28.84(35)% at 300 °C and increases to 51.57(71)% at 500 °C. The increase in the porosity of the TiO_2_-ZrO_2_ sol–gel coating with increasing sintering temperature can be attributed to the phase transformation and crystallization processes that occur as the temperature increases. As the temperature reaches 500 °C, the amorphous phase starts to crystallize, which can lead to an increase in porosity due to the rearrangement of atoms and the formation of new crystal structures [35]. This phase transformation and crystallization process can create more void spaces within the material, thus increasing its porosity. It should be added that the porosity of bone tissue varies widely among individuals, with values typically falling between 50% and 90%, reflecting the diverse states of bone health and structure across different people [69]. The TiO_2_-ZrO_2_ sol–gel coating applied to a Co-Cr-Mo surface and sintered at 500 °C was found to create a biomimetic structure with porosity levels similar to those of human bone tissue, suggesting it could effectively mimic the porous structure of bone in a living organism and potentially promote bone growth.

It should be noted that during sintering, the structure of TiO_2_-ZrO_2_ sol–gel coatings undergoes several changes that affect their properties. The primary changes include densification, crystallization, grain growth, and phase changes [70,71,72,73]. The coatings become denser as the sintering temperature increases. This densification process reduces the porosity of the coatings, which can enhance their mechanical and chemical properties [70,71]. Additionally, during sintering, the precursor used in the initial stage of applying TiO_2_-ZrO_2_ sol–gel coatings with an amorphous structure undergoes crystallization processes, and the structure changes from amorphous to crystalline, further affecting the surface morphology [70,71,72,73]. This change in structure can affect the optical and electrical properties of the coatings [70,71]. With the increasing sintering temperature, the grains within the coating grow larger. This grain growth can influence the mechanical properties and surface roughness of the coatings. Depending on the sintering conditions, the TiO_2_ and ZrO_2_ phases within the composite may undergo phase changes, which can further affect the overall properties of the coating. These structural changes are crucial for tailoring the properties of TiO_2_-ZrO_2_ sol–gel coatings to meet specific application requirements [70,71,72,73].

### 3.2. EDS Microanalysis of TiO_2_-ZrO_2_ Sol–Gel Coatings

**Figure 3:**
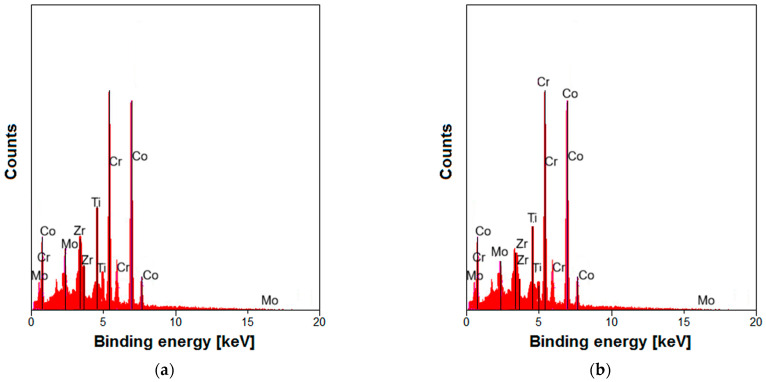
The EDS spectrum recorded from selected micro-region for a Co-Cr-Mo alloy with a TiO_2_-ZrO_2_ sol–gel coating sintered at (**a**) 300 °C and (**b**) 500 °C.

EDS microanalysis was used to study the effect of sintering temperature on the elemental composition of the TiO_2_-ZrO_2_ sol–gel coating on the Co-Cr-Mo alloy. The EDS spectrum, as indicated by Figure 3, was recorded from a specific micro-region of the coating surface. This spectrum shows the distribution of elements within the selected area, providing qualitative and quantitative information about the material’s composition. The EDS analysis confirms that Zr and Ti elements are present on the coating surface. Because of the low thickness of the TiO_2_-ZrO_2_ sol–gel coating, the chemical elements originating from the substrate (Co, Cr, and Mo) are also visible in the exemplary EDS spectra.

Table 1 quantifies the elemental composition, expressed in atomic percentage (at.%), for each sintering temperature of the TiO_2_-ZrO_2_ sol–gel coating on the Co-Cr-Mo substrate. Local measurements of the chemical composition of the tested materials using the EDS microanalytical method were given as mean values determined from measurements in five micro-areas. For the coating sintered at 300 °C, the elemental content is as follows: Co—33.81(29) at.%, Cr—26.33(71) at.%, Mo—3.55(20) at.%, Ti—20.71(39) at.%, and Zr—15.61(33) at.%. When sintered at 500 °C, the composition changes to the following: Co—36.42(40) at.%, Cr—29.53(69) at.%, Mo—5.64(21) at.%, Ti—18.21(51) at.%, and Zr—10.20(11) at.%. The numbers in parentheses represent the standard deviation associated with the measurement. The comparative EDS spectrum for the metallic substrate without any coating closely matched the expected composition of the Co-Cr-Mo alloy as specified by the manufacturer [8].

From the data provided, it is evident that the sintering temperature affects the elemental distribution on the surface of the TiO_2_-ZrO_2_ sol–gel coating. A higher sintering temperature causes less titanium and zirconium to be incorporated into TiO_2_-ZrO_2_ sol–gel coatings obtained from precursors present in the sol–gel solution. During the sintering process of TiO_2_-ZrO_2_ sol–gel coatings at high temperatures, the diffusion or volatilization of Ti and Zr can have significant implications on the final properties of the coating [67,70,74]. At high temperatures, the elements within the coating can undergo diffusion, which can affect the homogeneity and properties of the coating. Additionally, the volatilization of certain components can occur, leading to changes in the composition and structure of the coating. The SEM micrographs of ZrO_2_–TiO_2_ films at different sintering temperatures show that the microstructure of the coating changes with increasing temperature. This indicates that the diffusion of Ti and Zr atoms within the coating can lead to changes in the grain size and morphology, which are crucial for the mechanical and optical properties of the coating [67]. The high-temperature oxidation resistance of the sol–gel-derived ZrO_2_/(Al_2_O_3_–Y_2_O_3_) composite coating on a titanium alloy suggests that the stability of the coating at high temperatures is critical. The diffusion of Ti and Zr can affect the oxidation resistance by altering the protective oxide layer formed on the coating surface [74]. The corrosion behavior of amorphous sol–gel TiO_2_–ZrO_2_ nano-coatings annealed at 400 °C shows that the coating significantly improves corrosion resistance. This improvement is likely due to the formation of a stable oxide layer on the coating surface, which is influenced by the diffusion and volatilization of Ti and Zr during sintering [36]. The structural and surface chemical properties of sol–gel-derived TiO_2_–ZrO_2_ oxides with different Zr/Ti ratios indicate that the ratio of Ti to Zr can significantly affect the properties of the coating. Changes in the Zr/Ti ratio during sintering due to diffusion or volatilization can lead to variations in the coating’s properties [70]. The increase in the Ti/Zr ratio from 1.33 at 300 °C to 1.78 at 500 °C in the TiO_2_-ZrO_2_ sol–gel coating indicates that as the sintering temperature increases, there is a greater segregation of titanium into the coating compared to zirconium (Table 1). This suggests that at higher temperatures, the TiO_2_ component of the coating becomes more dominant, possibly due to differences in the thermal expansion coefficients or solubility of TiO_2_ and ZrO_2_, which can lead to the preferential incorporation of titanium into the crystalline structure or the formation of a more Ti-rich phase [75].

The depletion or redistribution of Ti and Zr in a TiO_2_-ZrO_2_ sol–gel coating sintered at high temperatures can significantly impact its functional properties, particularly corrosion resistance and biocompatibility [36,76,77,78,79]. The presence of TiO_2_ and ZrO_2_ in the coating enhances corrosion resistance due to their chemical stability and ability to form protective oxide layers on the surface of the substrate. The depletion or redistribution of Ti and Zr could lead to a reduction in the thickness or uniformity of these protective layers, thereby decreasing the overall corrosion resistance of the coating [36,76]. The Ti/Zr molar ratio plays a crucial role in determining the structural and chemical properties of the TiO_2_-ZrO_2_ composite. A change in this ratio due to depletion or redistribution could alter the phase composition and crystallinity of the coating, potentially affecting its corrosion resistance [77]. TiO_2_ and ZrO_2_ are known for their excellent biocompatibility, which is partly due to their inert nature and ability to form a stable oxide layer that minimizes direct contact with biological fluids. The depletion or redistribution of Ti and Zr could disrupt this protective layer, potentially leading to increased biological reactivity and reduced biocompatibility [78]. The equimolar TiO_2_-ZrO_2_ nanocomposite, with a predominant tetragonal phase for zirconia, has been shown to exhibit enhanced biocompatibility. Any alteration in the Ti/Zr ratio or phase composition due to depletion or redistribution could compromise these beneficial properties [79].

### 3.3. Surface Roughness Study

The geometric structure of the surface is an important factor determining the quality of a given dental appliance surface, which is the first to come into contact with the host tissues [80,81]. It can vary significantly depending on the specific design and surface modifications [82]. The quality of the surface of the material dedicated to the implant or prosthesis significantly affects the functional properties and durability of the element. Ensuring the appropriate roughness of the dental appliance surface limits the occurrence of health-threatening events during the long-term contact of the material with the aggressive and variable environment of the living organism. In practice, the most commonly used parameter determining the surface roughness of dental materials is Ra, which defines the arithmetical mean deviation of the assessed profile [83]. The Ra parameter provides stable results, not significantly influenced by scratches, contamination, or measurement noise, and is widely recognized for its resilience to these factors, ensuring consistent and reliable measurements in surface roughness analysis [84,85]. From literature data, it is evident that lower Ra values are generally preferred for dental materials to reduce bacterial adhesion and biofilm formation. This is because smoother surfaces are less conducive to bacterial attachment and biofilm accumulation, which can lead to dental caries and other oral health issues [86,87,88]. Additionally, a low Ra value limits the interaction of prosthetic elements with each other, which leads to longer failure-free use.

**Figure 4:**
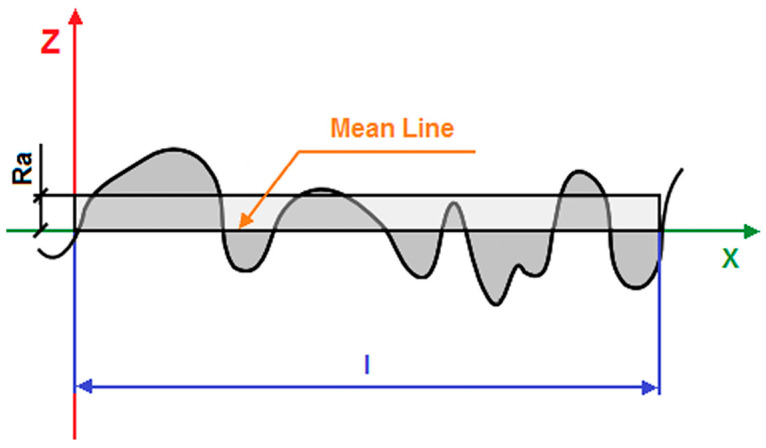
Graphical interpretation of surface roughness parameter Ra.

The Ra parameter refers to specific profile features and is a measure of surface roughness. Ra belongs to amplitude average parameters representing the arithmetic mean of the absolute ordinate Z(x) within the sampling length (l), as shown in Figure 4, and can be calculated based on Equation (2) [83]:

(2) $$Ra_{i}=\frac{1}{l}\int_{0}^{l}|Z(x)|dx.$$

Since the measured deviations Z_i_ from the x axis are of the order of a fraction of a millimeter, the Ra parameter is expressed in μm in practice. This parameter denotes the maximum allowable height of a rectangle whose surface area is equal to the surface area formed by the area between the graph of the Z(x) function and the x axis, where the x axis must pass through the Z(x) function so that the surface area of the Z(x) function located below the x axis is equal to the surface area located above the x axis.

**Figure 5:**
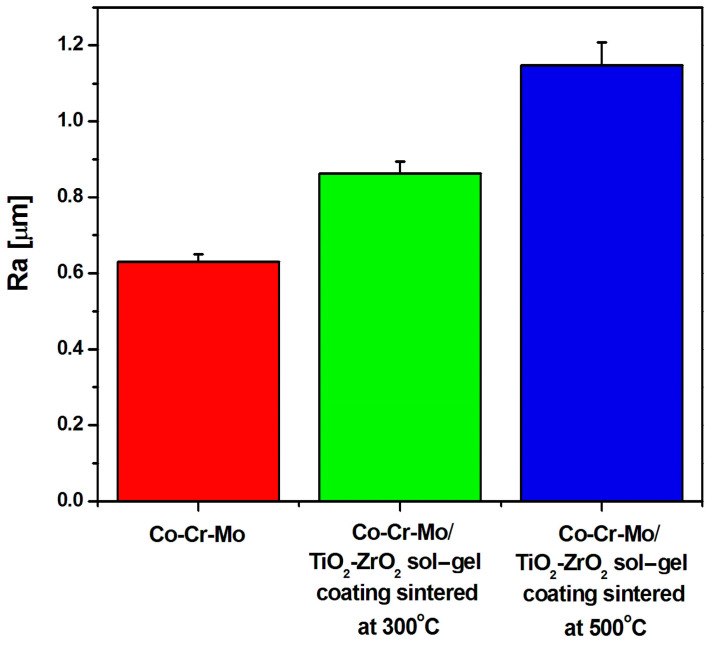
The surface roughness parameter Ra for the Co-Cr-Mo alloy in the absence and in the presence of the TiO_2_-ZrO_2_ sol–gel coating sintered at 300 °C and 500 °C.

The Ra measurement for the Co-Cr-Mo alloy was performed in the absence and in the presence of the TiO_2_-ZrO_2_ sol–gel coating sintered at 300 and 500 °C (Figure 5).

The Ra value for the Co-Cr-Mo alloy in its initial state is 0.630(20) µm, which indicates a smooth surface [89]. The Ra value for the TiO_2_-ZrO_2_ sol–gel coating sintered at 300 °C and 500 °C is 0.836(30) µm and 1.148(60) µm, respectively. The obtained results indicate that the sintered TiO_2_-ZrO_2_ coatings obtained on the Co-Cr-Mo alloy meet the requirements for metal alloys in dentistry regarding biocompatibility and the lowest possible Ra parameter value to limit the formation of bacterial biofilm [86]. The lower the surface roughness, the lower the risk of adhesion of *Streptococcus mutans* bacteria to the surface of prosthetic materials, which contribute to the formation of carious lesions [90]. A smooth surface also causes a change in the surface charge of solutions present in the oral cavity, as well as increased hydrophobicity [91].

The increase in surface roughness (Ra value) with the sintering temperature of TiO_2_-ZrO_2_ sol–gel coatings for dental implants has significant implications for osseointegration, bacterial adhesion, and patient comfort [92,93,94,95,96,97]. Surface roughness plays a crucial role in the initial bone apposition and subsequent osseointegration of dental implants. Studies have shown that moderately rough surfaces can enhance the mechanical interlocking between the implant and the surrounding bone, promoting better bone growth into surface irregularities [92]. An optimal roughness range is essential for maximizing the surface area available for bone contact without compromising the mechanical stability of the implant–bone interface. This range is typically considered to be between 1 and 2 µm for Ra [92]. Increased surface roughness can also affect bacterial adhesion. Smoother surfaces generally exhibit less bacterial adhesion compared to rougher surfaces, as the latter can provide more niches for bacteria to colonize [93]. Excessive roughness can therefore increase the risk of peri-implantitis, a common complication associated with dental implants [94]. The roughness of the implant surface can also influence patient comfort, particularly if the implant is in contact with soft tissues. A very rough surface may lead to soft tissue irritation and discomfort [95]. In areas where the implant is in close proximity to mucosal tissues, a smoother surface may be preferable to ensure patient comfort [96]. The obtained results indicate that the Ra value for the TiO_2_-ZrO_2_ sol–gel coating sintered at 500 °C aligns with the optimal range for dental applications (Figure 5).

### 3.4. In Vitro Electrochemical Tests Using the Open-Circuit Potential Method

The open-circuit potential method is a crucial technique used in in vitro corrosion resistance tests of dental materials [8,12,13,15,21,22,24,26,27,28,29,31,32,98,99,100]. This method is also known as open-circuit voltage, zero-current potential, corrosion potential, equilibrium potential, or rest potential [98]. It is often used to find the E_OC_ of a given electrochemical system, from which other experiments are based. The open-circuit potential method involves measuring the potential difference between a dental material as the WE and a RE in an electrolyte solution under open-circuit conditions. This provides information about the tendency of a material to corrode in the oral environment, as it reflects the approximate corrosion potential (E_cor_) of the material. Before starting the E_OC_ measurement, it is important to ensure that the surface of the dental material is stable and has reached a steady state. This is typically achieved by allowing the system to be undisturbed until the potential readings stabilize. The open-circuit potential method is often used in conjunction with other electrochemical techniques such as EIS and potentiodynamic polarization to provide a comprehensive understanding of the material’s corrosion resistance [5,6,8,99,100].

**Figure 6:**
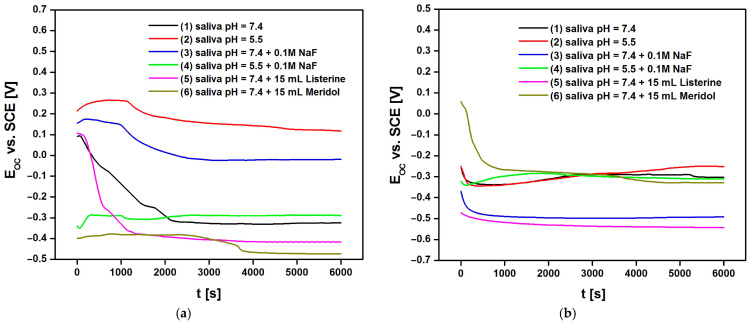
E_OC_—t dependence in unmodified and modified artificial saliva solutions at 37 °C for Co-Cr-Mo electrode with TiO_2_-ZrO_2_ sol–gel coating sintered at (**a**) 300 °C and (**b**) 500 °C.

Experimental data illustrating the dependence of E_OC_ on t for electrodes made of a Co-Cr-Mo alloy with a sol–gel layer sintered at 300 and 500 °C obtained in an artificial saliva solution without and with additives in the form of NaF and Listerine Total Care Teeth Protection^®^ by McNeil Consumer Healthcare McNeil-PPC, Inc., Fort Washington, PA, USA and Meridol^®^ by Colgate-Palmolive Company, New York, NY, USA mouthwashes are presented in Figure 6. Based on the obtained E_OC_ = f(t) curves in the applied corrosive environments, the tendency of each type of tested electrodes to corrode in the environment of unmodified and modified artificial saliva was determined.

The E_OC_ values provided in Table 2 indicate the corrosion behavior of a Co-Cr-Mo electrode with TiO_2_-ZrO_2_ sol–gel coatings sintered at different temperatures (300 and 500 °C) in various artificial saliva solutions at 37 °C after 6000 s of immersion in the electrolyte. E_OC_ is a measure of the electrode’s tendency to corrode in a given environment, with more negative values generally indicating a higher corrosion rate. From the data obtained, several trends can be observed. E_OC_ becomes more negative (indicating higher corrosion rates) when the pH of the saliva decreases from 7.4 to 5.5 for the unmodified saliva solutions—comparing electrolytes (1) and (2). The addition of 0.1M NaF to the saliva solutions significantly increases the corrosion rate (more negative E_OC_ values) for both sintering temperatures—electrolytes (3) and (4). The presence of Listerine Total Care Teeth Protection^®^ by McNeil Consumer Healthcare McNeil-PPC, Inc., Fort Washington, PA, USA and Meridol^®^ by Colgate-Palmolive Company, New York, NY, USA in the saliva solutions at pH 7.4 also increases the corrosion rate, with Listerine Total Care Teeth Protection^®^ by McNeil Consumer Healthcare McNeil-PPC, Inc., Fort Washington, PA, USA showing a stronger effect than Meridol^®^ by Colgate-Palmolive Company, New York, NY, USA for the 500 °C sintered coating—electrolytes (5) and (6). Overall, the coatings sintered at 500 °C tend to have more negative E_OC_ values, suggesting that they may be more susceptible to corrosion in these environments compared to those sintered at 300 °C. These results suggest that the corrosion behavior of the Co-Cr-Mo electrode with TiO_2_-ZrO_2_ sol–gel coatings is influenced by both the sintering temperature and the composition of the artificial saliva solution. The obtained results also indicate that the surface modification by applying TiO_2_-ZrO_2_ sol–gel coatings sintered at both 300 and 500 °C increases the corrosion resistance of the dental Co-Cr-Mo alloy in artificial saliva at physiological and acidic pH, which simulates inflammation in the body [8]. The E_OC_ values obtained after 6000 s of stabilization were treated as approximate E_cor_ values in further electrochemical measurements.

Various dental materials, including Co-Cr-Mo alloys and titanium-based materials, have been tested using the open-circuit potential method to assess their corrosion resistance in artificial saliva and other simulated oral environments [8,12,13,15,21,22,24,26,27,28,29,31,32,99,100]. It was found that E_OC_ is influenced by changes in the surrounding environment, such as temperature, pH, and the presence of different ions. This variability can make it challenging to compare results across different studies or to predict the material’s behavior in real-world conditions. While the open-circuit potential method provides information about the corrosion potential of a material, it does not offer detailed insights into the mechanisms of corrosion or the material’s resistance to localized corrosion, which are also critical for assessing the material’s long-term performance [15,100]. These limitations highlight the need for the careful control of experimental conditions and the use of complementary techniques to fully understand the corrosion behavior of dental materials.

### 3.5. In Vitro Electrochemical Tests Using Electrochemical Impedance Spectroscopy

Impedance tests were conducted to understand the corrosion mechanisms and kinetics at the electrode–electrolyte interface when the tested dental materials were exposed to unmodified and modified saliva solutions [8,26,27,29,99,100]. The electrode potential was stabilized until a steady E_OC_ was reached, as EIS requires a linear and steady-state system. The electrochemical corrosion resistance of metallic electrodes with a passive layer should be tested by the EIS method in the low-frequency range. This frequency range is particularly sensitive to the properties of the passive layer and the processes occurring at the electrode–electrolyte interface. The low-frequency range allows for the evaluation of the impedance behavior that is influenced by the capacitive properties of the passive layer, the charge transfer resistance across the interface, and the presence of any corrosion products or defects in the passive layer.

**Figure 7:**
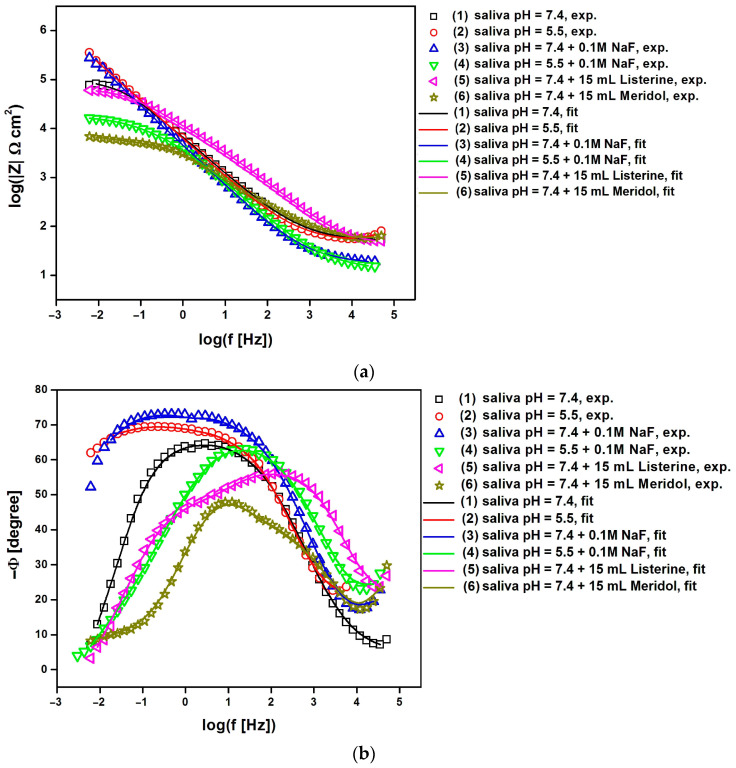
Experimental (symbols) and simulated (lines) Bode diagrams for a Co-Cr-Mo electrode coated with a TiO_2_-ZrO_2_ sol–gel coating sintered at 300 °C in artificial saliva solution before and after modification at 37 °C: (**a**) impedance module; (**b**) phase angle shift.

**Figure 8:**
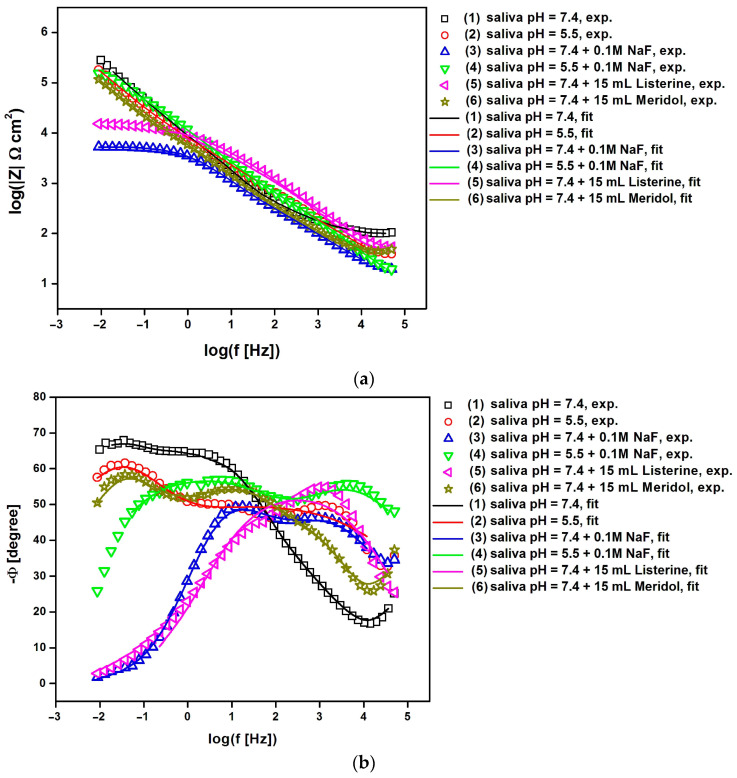
Experimental (symbols) and simulated (lines) Bode diagrams for a Co-Cr-Mo electrode coated with a TiO_2_-ZrO_2_ sol–gel coating sintered at 500 °C in artificial saliva solution before and after modification at 37 °C: (**a**) impedance module; (**b**) phase angle shift.

The EIS analysis involved the use of Bode diagrams to compare experimental and simulated data for Co-Cr-Mo electrodes coated with TiO_2_-ZrO_2_ sol–gel coatings sintered at 300 °C (Figure 7) and 500 °C (Figure 8) obtained under potentiostatic control at E_OC_ in artificial saliva solution at different pH levels and in the presence of additives such as NaF, Listerine Total Care Teeth Protection^®^ by McNeil Consumer Healthcare McNeil-PPC, Inc., Fort Washington, PA, USA, and Meridol^®^ by Colgate-Palmolive Company, New York, NY, USA.

The decreasing value of the logarithm of the impedance modulus at the lowest frequency in Figure 7a and Figure 8a is indicative of a decline in the corrosion resistance of the material, potentially due to changes in the passive layer or the electrochemical environment at the interface. The impedance modulus is a measure of how much the system opposes the flow of current, and it is influenced by the properties of the material and the electrolyte solution [8,26,27,29,99,100]. The impedance modulus at low frequencies is particularly sensitive to the capacitive properties of the passive layer formed by the coating and the charge transfer resistance at the electrode–electrolyte interface. When the logarithm of the impedance modulus decreases at low frequencies, it suggests that the passive layer may be less effective in protecting the underlying material from corrosion. This could be due to the presence of defects in the coating, the dissolution of the coating material, or the formation of less protective corrosion products. The decrease in the impedance modulus can also be related to a reduction in the charge transfer resistance, which is a measure of the ease with which ions can be exchanged at the interface, indicating a more active corrosion process.

In the case of the Co-Cr-Mo electrode with a TiO_2_-ZrO_2_ sol–gel coating sintered at 300 °C, the highest value of the logarithm of the impedance modulus at the lowest tested frequency f = 10 mHz equal to 5.32(79) Ω cm^2^ was recorded in the artificial saliva environment with neutral pH and NaF (3) addition, which indicates the highest corrosion resistance among all the electrolytes used from (1) to (6), as shown in Figure 6a. The lowest log|Z| value of 3.82(56) Ω cm^2^ for the same electrode type was obtained in artificial saliva solution at pH = 7.4 modified with Meridol^®^ by Colgate-Palmolive Company, New York, NY, USA mouthwash (6), which proves the strong influence of the additive in the form of ready-to-use Meridol by Colgate-Palmolive Company, New York, NY, USA mouthwash on the corrosion resistance of the surface-modified Co-Cr-Mo electrode in artificial saliva. The increase in the sintering temperature of the TiO_2_-ZrO_2_ sol–gel coating up to 500 °C affects the corrosion resistance of the Co-Cr-Mo electrode (Figure 8a). In the case of the Co-Cr-Mo electrode with a TiO_2_-ZrO_2_ sol–gel coating sintered at 500 °C, the highest value of log|Z|_f = 10 mHz_ equal to 5.45(71) Ω cm^2^ was recorded in the environment of unmodified artificial saliva at pH = 7.4 (1), which indicates the highest corrosion resistance determined in electrolytes from (1) to (6). The lowest corrosion resistance for the same type of electrode was found based on the lowest log|Z| value of 3.72(47) Ω cm^2^ in artificial saliva solution at pH = 7.4 with the addition of 0.1M NaF (3). It should be emphasized that the log|Z| values obtained at the lowest frequency f = 10 mHz indicate an increase in the corrosion resistance of the Co-Cr-Mo electrode covered with TiO_2_-ZrO_2_ sol–gel coatings sintered both at 300 and 500 °C in comparison with the log|Z|_f = 10 mHz_ values obtained for the Co-Cr-Mo electrode without surface modification in artificial saliva at pHs of 7.4 and 5.5 [8]. The slope of all curves showing the relationship log|Z| = log(f) in Figure 7a and Figure 8a in the mid-frequency range is close to —1.

The shape of the EIS spectra shown in Figure 7b and Figure 8b is characteristic of passivated metallic materials undergoing pitting corrosion in a biological environment containing aggressive chloride ions [8,26,27,29,99,100]. In the case of the Co-Cr-Mo electrode covered with TiO_2_-ZrO_2_ sol–gel coatings sintered at 300 °C, one time constant is visible in the circuit in solutions (1) to (6) in Figure 7b. In Figure 8b, a similar impedance behavior is seen for the Co-Cr-Mo electrode coated with a TiO_2_-ZrO_2_ sol–gel coating sintered at 500 °C in a neutral pH artificial saliva solution (1) and with the addition of ready-to-use Listerine Total Care Teeth Protection^®^ by McNeil Consumer Healthcare McNeil-PPC, Inc., Fort Washington, PA, USA mouthwash (5). The presence of only one time constant in the circuit indicates that there is a single frequency response in the system, suggesting that the corrosion process involves a single dominant mechanism or process under these conditions [8,26,29,99,100]. In the remaining tested corrosive environments (2), (3), (4), and (6), two time constants are observed in the circuit obtained for the Co-Cr-Mo electrode covered with TiO_2_-ZrO_2_ sol–gel coatings sintered at 500 °C (Figure 8b). The obtained Bode plots show two distinct regions of frequency response, suggesting that the corrosion process involves two distinct mechanisms or processes under these conditions [100].

**Figure 9:**
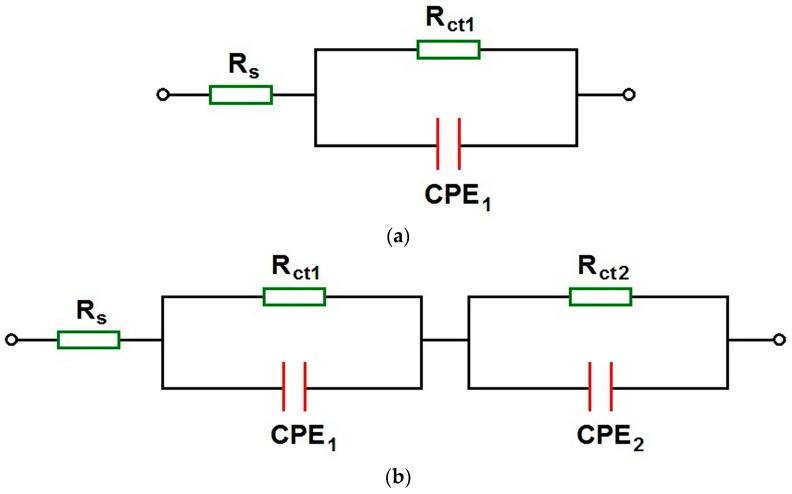
An equivalent electrical circuit for the Co-Cr-Mo electrode|TiO_2_-ZrO_2_ sol–gel coating|saliva solution in the pitting corrosion process: (**a**) 1CPE model and (**b**) 2CPE model.

To understand the detailed mechanism and kinetics of corrosion processes for the Co-Cr-Mo electrode covered with TiO_2_-ZrO_2_ sol–gel coatings sintered at 300 and 500 °C in the artificial saliva environment before and after the modification of its chemical composition, the obtained EIS experimental data were approximated using appropriate electrical equivalent circuits (Figure 9).

The simplest electrical equivalent circuit in the form of the modified Randles circuit was used to model the experimental impedance spectra with one time constant. This circuit consists of a solution resistance (R_s_) in series with a parallel combination of double-layer capacitance (C_dl_) and charge transfer resistance (R_ct_). As the capacitance often differs from the ideal due to the roughness of the electrode surface, C_dl_ was represented by a constant phase element (CPE). The CPE’s impedance is defined by Equation (3):

(3) $${\hat{Z}}_{CPE}=\frac{1}{T{\left(j\omega \right)}^{\;\phi }}$$

where T is the capacitance parameter, dependent on the electrode potential, and ϕ is related to the deviation from purely capacitive behavior [63]. When ϕ equals 1, the CPE behaves like a pure capacitor, and T equals C_dl_. Other values of f represent different behaviors, such as Warburg impedance, resistance, or inductance. This so-called 1CPE model includes one semicircle on the complex plane plot, characterized by R_s_, T_1_, ϕ_1_, and R_ct1_ (Figure 9a). This model is commonly used to assess the corrosion resistance of passivated metallic electrodes in simulated body fluids [8,26,29,99,100].

For the experimental impedance spectra with two time constants, a more complex model was used to study the interfacial properties of the electrochemical system (Figure 9b). This so-called 2CPE model consists of two Randles circuits in series, producing two semicircles on the complex plane plot. The high-frequency semicircle is described by R_s_, T_1_, ϕ_1_, and R_ct1_. The low-frequency semicircle described by T_2_, ϕ_2_, and R_ct2_. The use of two time constants in the 2CPE model suggests a more complex electrochemical behavior, possibly due to the presence of multiple layers or processes at the electrode surface [100]. The resistance values (R_s_, R_ct1_, and R_ct2_) are generally higher in the 2CPE model as compared with those in the 1CPE model, indicating a more detailed representation of the corrosion protection offered by the coating.

The average double-layer capacitance (${\bar{C}}_{dl}$) of the corrosion process was determined from the Brug relationship described by Equation (4) [101]:

(4) $$T={\bar{C}}_{dl}^{\phi }{\left(R_{s}^{-1}+R_{ct}^{-1}\right)}^{1-\phi }$$

Bode diagrams in Figure 7 and Figure 8 confirm the very good fit of the experimental EIS data to the theoretical data. Table 3 presents the equivalent electrical circuit parameters obtained from EIS experiments for Co-Cr-Mo electrodes coated with TiO_2_-ZrO_2_ sol–gel coatings sintered at 300 °C and exposed to various artificial saliva environments at 37 °C. The comparative fitting parameters for the Co-Cr-Mo electrodes coated with TiO_2_-ZrO_2_ sol–gel coatings sintered at 500 °C are presented in Table 4 and Table 5. The detailed error in determining the fitting parameters was below 15%.

For the Co-Cr-Mo electrode with the TiO_2_-ZrO_2_ sol–gel coating sintered at 300 °C (Table 3), the data show that R_s_ varies with the electrolyte type, with the lowest values observed in the presence of 0.1M NaF, regardless of the pH level. The T_1_ values are generally similar across different electrolytes, with slightly higher values in the presence of NaF. The ϕ1 values are close to 0.75, suggesting a non-ideal capacitive behavior. R_ct1_ is the highest in the presence of 0.1M NaF, indicating a more significant barrier to electron transfer. The ${\bar{C}}_{dl}$ varies, with the highest value observed in the saliva with added NaF at pH 7.4.

For the Co-Cr-Mo electrode with the TiO_2_-ZrO_2_ sol–gel coating sintered at 500 °C (Table 4 and Table 5), R_s_ is lower compared to that of the coating sintered at 300 °C, indicating a more conductive electrolyte environment. The T_1_ values are lower, and the ϕ_1_ values are close to 0.75, similar to the electrode with the coating sintered at 300 °C. R_ct1_ is significantly higher in the unmodified saliva at pH 7.4, suggesting a more protective coating.

When using the 2CPE model (Table 5), which includes two time constants, the data show that the second CPE (T_2_, ϕ_2_) and the second charge transfer resistance (R_ct2_) are introduced. The T_2_ values are lower than T_1_, and the ϕ_2_ values are around 0.55–0.57, indicating a different type of non-ideal capacitive behavior. The R_ct2_ values are lower than R_ct1_, suggesting a less significant barrier to electron transfer associated with the second time constant. The ${\bar{C}}_{dl}$ values are lower compared to the electrode with the TiO_2_-ZrO_2_ sol–gel coating sintered at 300 °C, indicating a possible difference in the electrode’s surface properties. The obtained EIS data suggest that the sintering temperature and the composition of the artificial saliva environment affect the electrochemical properties of the Co-Cr-Mo electrodes with TiO_2_-ZrO_2_ sol–gel coatings. The addition of NaF and the use of mouthwashes like Listerine Total Care Teeth Protection^®^ by McNeil Consumer Healthcare McNeil-PPC, Inc., Fort Washington, PA, USA and Meridol^®^ by Colgate-Palmolive Company, New York, NY, USA significantly alter the electrochemical behavior, with NaF generally increasing the charge transfer resistance and the double-layer capacitance, indicating a more protective environment for the electrode. The EIS data for the unmodified Co-Cr-Mo obtained under the same electrochemical conditions also revealed the effect of pH on the uncoated Co-Cr-Mo electrode [8]. It was found that at pH 7.4, the resistance R_1_ is higher than that at pH 5.5, indicating better corrosion resistance at a higher pH. The addition of 0.1 M NaF increases R_1_ at both pH levels, suggesting improved corrosion resistance in the presence of fluoride ions. The T_1_ values are lower at pH 7.4 compared to pH 5.5, which may indicate a more compact or less defective passive layer at higher pH, and the presence of Listerine Total Care Teeth Protection^®^ by McNeil Consumer Healthcare McNeil-PPC, Inc., Fort Washington, PA, USA and Meridol^®^ by Colgate-Palmolive Company, New York, NY, USA mouthwashes significantly increases R_2_, suggesting a protective effect on the electrode surface.

R_ct_ is a measure of the electron transfer kinetics at the electrode–electrolyte interface. A higher R_ct_ value indicates slower electron transfer, which is generally associated with better corrosion resistance [5,6,8,26,29,47,63,99,100,101]. If the R_ct_ values increase for coatings in NaF and mouthwash environments, this suggests that the coatings exhibit better corrosion resistance in these conditions [8,26,45,46,47,48]. ${\bar{C}}_{dl}$ is related to the interfacial properties and the roughness of the surface [5,6,8,26,29,47,63,99,100,101]. A lower ${\bar{C}}_{dl}$ value typically indicates a smoother surface, which can be beneficial for corrosion resistance as it reduces the available surface area for corrosion reactions. The structural differences observed at different sintering temperatures of TiO_2_-ZrO_2_ sol–gel coatings can influence EIS parameters. For instance, higher sintering temperatures may lead to denser coatings with fewer defects, which can result in higher R_ct_ values. Conversely, lower sintering temperatures may produce less dense coatings with lower R_ct_ values (see Figure 2, Table 3, Table 4 and Table 5). An increase in R_ct_ for coatings in NaF and mouthwash environments suggests a long-term benefit in terms of corrosion resistance. However, it is important to consider other factors such as the mechanical properties of coatings. If the increased R_ct_ is accompanied by a decrease in mechanical strength, this could potentially lead to issues such as delamination or degradation over time. The observed EIS trends are consistent with known models of corrosion mechanisms in metal oxides in which the formation of a protective oxide layer on the metallic surface leads to increased R_ct_ values [5,6,8,47,99,100,101].

### 3.6. In Vitro Cyclic Potentiodynamic Polarization Curve Study

**Figure 10:**
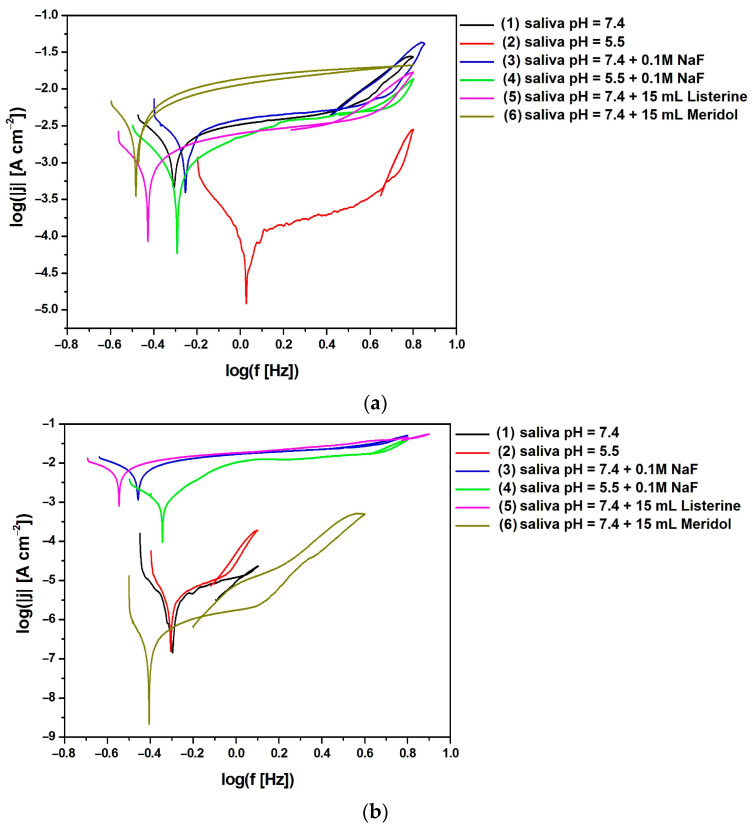
The cyclic potentiodynamic polarization curve at a polarization scan rate of v = 1 mV s^−1^ in the unmodified and modified saliva solutions at 37 °C for the Co-Cr-Mo electrode covered with the TiO_2_-ZrO_2_ sol–gel coating sintered at (**a**) 300 °C and (**b**) 500 °C.

The susceptibility to pitting corrosion of the Co-Cr-Mo electrode covered with the TiO_2_-ZrO_2_ sol–gel coating sintered at 300 and 500 °C in the unmodified and modified saliva solutions at 37 °C was determined by cyclic potentiodynamic polarization curve tests (Figure 10).

The key parameters for corrosion resistance include the corrosion potential (E_cor_), corrosion current density (j_cor_), protection potential (E_p_), and breakdown potential (E_bd_). The difference between E_bd_ and E_p_ (E_bd_—E_p_) denoting the width of the hysteresis loop in the anodic curves shown in Figure 10 is also an important indicator of the susceptibility to pitting corrosion.

For the Co-Cr-Mo electrode covered with the TiO_2_-ZrO_2_ sol–gel coating sintered at 300 °C (Table 6), the lowest corrosion current density (indicating higher corrosion resistance) is observed in the unmodified saliva solution with pH = 7.4, followed by the solution with added NaF. The highest corrosion current density is seen in the saliva solution with pH = 5.5, suggesting that acidic conditions increase the susceptibility to corrosion. The addition of NaF generally improves the corrosion resistance, as evidenced by lower j_cor_ values. The presence of Listerine Total Care Teeth Protection^®^ by McNeil Consumer Healthcare McNeil-PPC, Inc., Fort Washington, PA, USA and Meridol^®^ by Colgate-Palmolive Company, New York, NY, USA also affects the corrosion behavior, with Meridol^®^ by Colgate-Palmolive Company, New York, NY, USA showing a significant negative shift in E_cor_ and a high E_bd_—E_p_ value, indicating a protective effect.

In the case of the Co-Cr-Mo electrode covered with the TiO_2_-ZrO_2_ sol–gel coating sintered at 500 °C (Table 7), the corrosion current densities are generally lower than those for the electrode with the coating sintered at 300 °C, indicating better corrosion resistance. The lowest j_cor_ is observed in the unmodified saliva solution with pH = 7.4, followed by the solution with added NaF. The acidic saliva solution (pH = 5.5) again shows higher corrosion current density, confirming the detrimental effect of acidity. The addition of NaF and Listerine Total Care Teeth Protection^®^ by McNeil Consumer Healthcare McNeil-PPC, Inc., Fort Washington, PA, USA improves corrosion resistance, while that of Meridol^®^ by Colgate-Palmolive Company, New York, NY, USA shows a significant negative shift in E_cor_ and a high E_bd_—E_p_ value, similar to the electrode sintered at 300 °C, suggesting a protective effect. Based on the obtained results, it can be concluded that the produced TiO_2_-ZrO_2_ coatings have an impact on reducing the susceptibility to pitting corrosion of the Co-Cr-Mo dental alloy in the oral cavity environment with physiological and acidic pH [8]. According to the literature, Co-Cr-Mo alloys exhibit varying degrees of corrosion resistance depending on the processing method, surface modification, and the conditions of exposure [8,26,27,29,30,32].

The results of cyclic potentiodynamic polarization curves indicating reduced pitting susceptibility for TiO_2_-ZrO_2_ sol–gel coatings sintered at 500 °C can be attributed to several mechanistic factors. To delve deeper into these reasons, it is essential to consider microstructural changes, the role of micro-cracks, and the influence of environmental factors such as pH and the composition of artificial saliva. Sintering at higher temperatures can lead to increased densification and grain growth within coatings. This can result in a more homogeneous and compact structure, which is generally more resistant to corrosion (see Figure 2). Micro-cracks can form in coatings due to thermal stresses during sintering, which can actually enhance passivation properties. This is because they can provide pathways for the electrolyte to access the underlying metal, promoting the formation of a more uniform and protective oxide layer. The pH and the composition of artificial saliva can significantly influence the initiation of pitting corrosion [8,26,45,46,47,48]. A more neutral or slightly alkaline pH can favor the formation of a stable passivation layer, whereas a more acidic environment can lead to its breakdown. The presence of certain ions (e.g., chloride, fluoride) can also affect the stability of the passivation layer and the propensity for pitting corrosion. The composition of artificial saliva can provide buffering effects that stabilize the pH at the coating surface, which can be beneficial for maintaining the integrity of the passivation layer [102].

The comparison between the effects of different artificial saliva components on TiO_2_-ZrO_2_ coatings is crucial for understanding their performance in real-world dental applications. These components, such as NaF and various mouthwash formulations, can interact with the coatings at the molecular level, influencing their corrosion resistance and overall stability. Fluoride ions can interact with TiO_2_-ZrO_2_ coatings by forming fluoride-rich oxide layers. These layers are generally more stable and protective than the native oxide layers, providing enhanced corrosion resistance. Fluoride ions can replace hydroxide ions in the oxide layer, forming compounds such as TiF_4_ and ZrF_4_, which are highly resistant to corrosion [103]. The presence of fluoride ions can lead to the formation of a more stable and uniform passivation layer, reducing the susceptibility to pitting corrosion [8,26,45,46,47,48]. Fluoride-rich oxide layers can reduce the release of metal ions from the coatings, further enhancing their stability in corrosive environments [102]. Mouthwashes contain a variety of components, including alcohol, chlorhexidine, essential oils, and other antimicrobial agents. These components can have different effects on TiO_2_-ZrO_2_ coatings. Alcohol is often used as a solvent and antimicrobial agent in mouthwashes. It can influence the wetting properties of the coatings and potentially affect the stability of the passivation layer. Alcohol can reduce the surface tension of saliva, affecting the adhesion and cohesion of the passivation layer [104]. The effects of mouthwash components on corrosion resistance can be variable, depending on the specific formulation and concentration of the active ingredients [105]. Some mouthwash components, particularly those with high alcohol content or strong antimicrobial agents, may disrupt the passivation layer, potentially increasing the susceptibility to corrosion [105]. NaF generally enhances the stability of the passivation layer, providing better long-term corrosion resistance. Mouthwashes, depending on their composition, may have variable effects, ranging from beneficial to potentially disruptive. For dental implants, the use of NaF-containing products can be beneficial for maintaining the integrity of the coatings. However, the choice of mouthwash should be considered, as some formulations may affect the stability of the passivation layer [8].

The investigated TiO_2_-ZrO_2_ coatings provide significant protection in both physiological and acidic conditions. The underlying mechanisms can be attributed to the unique properties of the dual composition and its interaction with the environment at different pH levels. In physiological conditions, both TiO_2_ and ZrO_2_ form stable oxide layers that protect the underlying metal from corrosion. The stability of these layers is due to the formation of strong metal–oxygen bonds [106]. In acidic conditions, the stability of the oxide layers can be challenged due to the increased level of activity of hydrogen ions. However, the dual composition of TiO_2_-ZrO_2_ can mitigate this effect by forming mixed oxide layers that are more resistant to acidic attack [107]. The combination of TiO_2_ and ZrO_2_ can lead to a synergistic effect where the formation of a mixed oxide layer provides better protection than either oxide alone. This mixed layer can offer enhanced passivation properties, reducing the susceptibility to corrosion in both physiological and acidic conditions. The dual composition can also improve the mechanical properties of the coatings, making them more resistant to crack propagation and delamination, which are common issues in corrosive environments [108]. At physiological pH, the oxide layers formed by both TiO_2_ and ZrO_2_ are stable and protective. The formation of a compact and adherent oxide layer prevents the ingress of corrosive species [102]. In acidic conditions, the increased concentration of hydrogen ions can lead to the partial dissolution of the oxide layer. However, the dual composition of TiO_2_-ZrO_2_ can resist this dissolution better than either oxide alone. The mixed oxide layer can repel hydrogen ions more effectively, maintaining its integrity even in acidic environments [103]. The ability of TiO_2_-ZrO_2_ coatings to provide significant protection in both physiological and acidic conditions is clinically significant for several reasons. The oral environment can experience fluctuations in pH due to dietary habits, oral hygiene practices, and the presence of certain bacteria [109]. The robustness of these coatings ensures consistent protection against corrosion under varying conditions [105]. The resistance of these coatings to both physiological and acidic conditions can lead to an improved longevity of dental implants, reducing the need for replacement and minimizing patient discomfort [110].

To provide a comprehensive evaluation of TiO_2_-ZrO_2_ coatings, it is essential to compare them with other commonly used coatings such as pure TiO_2_, ZrO_2_, or hydroxyapatite. Additionally, discussing the expected long-term performance based on accelerated aging tests or literature evidence will help appreciate the practical applicability of these coatings in dental alloys. The dual composition of TiO_2_-ZrO_2_ generally exhibits better corrosion resistance compared to pure TiO_2_ or ZrO_2_ coatings. This is due to the formation of a more stable and protective mixed oxide layer [107]. TiO_2_-ZrO_2_ coatings often show higher durability and resistance to corrosion compared to hydroxyapatite coatings, which can degrade over time in physiological environments [106]. The combination of TiO_2_ and ZrO_2_ can lead to improved mechanical properties, including higher toughness and resistance to crack propagation [108]. TiO_2_-ZrO_2_ coatings typically exhibit better mechanical strength and wear resistance compared to hydroxyapatite coatings [102]. Both TiO_2_ and ZrO_2_ are known for their excellent biocompatibility. The dual composition maintains this biocompatibility while offering additional benefits in terms of corrosion resistance and mechanical properties [103]. Hydroxyapatite coatings are highly biocompatible and osteoconductive. TiO_2_-ZrO_2_ coatings, while not osteoconductive, are also biocompatible and can be designed to support osseointegration [110].

Accelerated aging tests can be performed by exposing the obtained TiO_2_-ZrO_2_ coatings to simulated oral environments for extended periods. These tests can help predict the long-term stability and performance of the coatings [105]. Combining mechanical stress (e.g., cyclic loading) with chemical stress (e.g., acidic and alkaline solutions) can provide a more comprehensive understanding of the coatings’ durability [107]. Studies on similar coatings have shown that TiO_2_-ZrO_2_ coatings can maintain their integrity and protective properties over extended periods in physiological environments [106]. The resistance of TiO_2_-ZrO_2_ coatings to degradation in both acidic and alkaline conditions suggests that they can withstand the varying pH of the oral environment over time [103].

The obtained TiO_2_-ZrO_2_ sol–gel coatings show excellent adherence, flexibility, and compactness, which are crucial for effective corrosion protection. They are relatively thin and are capable of forming a protective barrier against corrosive environments. The TiO_2_-ZrO_2_ sol–gel coatings provide enhanced corrosion resistance by creating a barrier that prevents the ingress of corrosive species. This is particularly important for dental appliances where the alloy is exposed to saliva and other biological fluids. The sol–gel method allows for the incorporation of various metal oxides into the coating, which can further enhance the properties of the coating. Further research should focus on the precise fabrication of nanostructured mixed metal oxides using the sol–gel method, which can further improve the mechanical and protective properties of the coatings.

Translating the findings on TiO_2_-ZrO_2_ sol–gel coatings into clinical practice involves several critical steps, including scaling up the technology, addressing regulatory hurdles, ensuring biocompatibility, and optimizing manufacturing processes. The sol–gel process used to create these coatings needs to be optimized for large-scale production. This includes standardizing the precursor solutions, coating parameters (e.g., thickness, uniformity), and sintering conditions [111]. Implementing rigorous quality control measures is essential to ensure the consistency and reliability of the coatings. This includes monitoring the coating thickness, adhesion, and corrosion resistance [105]. Before clinical use, the coatings must undergo preclinical testing to evaluate their safety and efficacy. This includes in vitro tests (e.g., cytotoxicity, biocompatibility) and in vivo animal studies [112]. Successful preclinical testing should be followed by clinical trials to assess the performance of the coatings in human subjects. These trials are crucial for gaining regulatory approval [113]. The coatings must be tested for cytotoxicity and biocompatibility using standardized methods. This includes assessing the effects of the coatings on cell viability, proliferation, and differentiation [114]. Evaluating the immunological response to the coatings is also important to ensure they do not induce adverse reactions in the body [115]. The sol–gel process must be optimized for industrial manufacturing, ensuring that the coatings can be produced consistently and efficiently [111]. Automating the coating process can improve efficiency and reduce variability. This includes automating the application of the sol–gel solution, drying, and sintering steps [51]. The coatings must be compatible with the dental alloys commonly used in implants and restorations. This includes ensuring adhesion and mechanical compatibility [102]. Dentists and oral surgeons need to be trained in the application and handling of implants and restorations coated with TiO_2_-ZrO_2_ [110]. The potential of TiO_2_-ZrO_2_ sol–gel coatings into clinical practice requires a careful consideration of scaling up the technology, addressing regulatory hurdles, ensuring biocompatibility, and optimizing manufacturing processes. By following standardized testing protocols and adhering to regulatory guidelines, these coatings can be successfully integrated into dental restorations, offering improved longevity and performance.

## 4. Conclusions

The applied sol–gel method allows us to obtain thin TiO_2_-ZrO_2_ coatings on the surface of the Co-Cr-Mo dental alloy (Bego Wirobond^®^ C by BEGO Bremer Goldschlägerei Wilh. Herbst GmbH & Co. KG, Bremen, Germany), minimizing material waste due to precise and accurate synthesis, which reduces the amount of raw materials used in the process. SEM analysis shows that sintering temperatures at 300 and 500 °C significantly affect the coatings’ morphology and structure, with higher temperatures causing micro-cracks but increasing density. EDS confirms elemental composition changes with temperature, potentially due to volatilization or diffusion. Roughness measurements indicate that the coatings meet dental requirements for biocompatibility and bacterial resistance. Electrochemical tests reveal that the coatings enhance corrosion protection, particularly at higher sintering temperatures and in the presence of NaF and mouthwashes (Listerine Total Care Teeth Protection^®^ by McNeil Consumer Healthcare McNeil-PPC, Inc., Fort Washington, PA, USA, Meridol^®^ by Colgate-Palmolive Company, New York, NY, USA), suggesting their suitability for various oral environments. These coatings are effective in reducing pitting corrosion susceptibility, critical for the longevity and safety of dental devices. The findings underscore the potential of TiO_2_-ZrO_2_ sol–gel coatings in improving the performance of Co-Cr-Mo dental alloys in various oral environments.

## Figures and Tables

**Table 1:** Elemental content on surface of Co-Cr-Mo alloy with TiO_2_-ZrO_2_ sol–gel coating sintered at 300 °C and 500 °C.

Sintering Temperature [°C]	Element Content [at.%]				
	Co	Cr	Mo	Ti	Zr
300	33.81(29)	26.33(71)	3.55(20)	20.71(39)	15.61(33)
500	36.42(40)	29.53(69)	5.64(21)	18.21(51)	10.20(11)

**Table 2:** E_OC_ for the Co-Cr-Mo electrode with the TiO_2_-ZrO_2_ sol–gel coating sintered at 300 and 500 °C in unmodified and modified artificial saliva solutions at 37 °C after 6000 s.

Electrolyte Type	E_OC_ [V]	
	TiO_2_-ZrO_2_ Sol–Gel Coating Sintered at 300 °C	TiO_2_-ZrO_2_ Sol–Gel Coating Sintered at 500 °C
(1) Saliva with pH = 7.4	−0.207(17)	−0.256(13)
(2) Saliva with pH = 5.5	0.023(10)	−0.242(19)
(3) Saliva with pH = 7.4 + 0.1M NaF	−0.020(14)	−0.492(27)
(4) Saliva with pH = 5.5 + 0.1M NaF	−0.291(16)	−0.344(24)
(5) Saliva with pH = 7.4 + 15 mL Listerine Total Care Teeth Protection^®^ by McNeil Consumer Healthcare McNeil-PPC, Inc., Fort Washington, PA, USA	−0.417(23)	−0.543(33)
(6) Saliva with pH = 7.4 + 15 mL Meridol^®^ by Colgate-Palmolive Company, New York, NY, USA	−0.474(23)	−0.328(25)

**Table 3:** Equivalent electrical circuit parameters obtained by fitting EIS experimental data using the 1CPE model for a Co-Cr-Mo electrode with a TiO_2_-ZrO_2_ sol–gel coating sintered at 300 °C in an unmodified and modified artificial saliva environment at 37 °C.

Artificial Saliva Solution	R_s_ [Ω cm^2^]	T_1_ [F cm^−2^ s^ϕ−1^]	ϕ_1_	R_ct1_ [Ω cm^2^]	${\bar{C}}_{dl}$ [F cm^−2^]
(1) pH = 7.4	55(8)	3.66(55)·10^−5^	0.74(11)	1.01(15)·10^5^	3.88·10^−3^
(2) pH = 5.5	58(8)	3.79(57)·10^−5^	0.76(11)	3.21(48)·10^5^	2.79·10^−3^
(3) pH = 7.4 + 0.1M NaF	20(3)	4.70(71)·10^−5^	0.72(10)	8.21(23)·10^5^	1.62·10^−2^
(4) pH = 5.5 + 0.1M NaF	14(2)	6.63(92)·10^−5^	0.71(10)	1.64(25)·10^4^	1.65·10^−3^
(5) pH = 7.4 + 15 mL Listerine Total Care Teeth Protection^®^ by McNeil Consumer Healthcare McNeil-PPC, Inc., Fort Washington, PA, USA	31(4)	2.32(35)·10^−5^	0.63(9)	6.64(96)·10^4^	2.92·10^−3^
(6) pH = 7.4 + 15 mL Meridol^®^ by Colgate-Palmolive Company, New York, NY, USA	47(7)	8.79(99)·10^−5^	0.84(12)	7.14(99)·10^3^	3.50·10^−2^

**Table 4:** Equivalent electrical circuit parameters obtained by fitting EIS experimental data using the 1CPE model for a Co-Cr-Mo electrode with a TiO_2_-ZrO_2_ sol–gel coating sintered at 500 °C in an unmodified and modified artificial saliva environment at 37 °C.

Artificial Saliva Solution	R_s_ [Ω cm^2^]	T_1_ [F cm^−2^ s^ϕ−1^]	ϕ_1_	R_ct_ [Ω cm^2^]	${\bar{C}}_{dl}$ [F cm^−2^]
(1) pH = 7.4	45(6)	2.26(34)·10^−5^	0.74(10)	2.34(35)·10^6^	4.08·10^−3^
(5) pH = 7.4 + 15 mL Listerine Total Care Teeth Protection ^®^ by McNeil Consumer Healthcare McNeil-PPC, Inc., Fort Washington, PA, USA	25(4)	1.71(26)·10^−5^	0.78(11)	1.50(23)·10^4^	1.57·10^−3^

**Table 5:** Equivalent electrical circuit parameters obtained by fitting EIS experimental data using the 2CPE model for a Co-Cr-Mo electrode with a TiO_2_-ZrO_2_ sol–gel coating sintered at 500 °C in an unmodified and modified artificial saliva environment at 37 °C.

Artificial Saliva Solution	R_s_ [Ω cm^2^]	T_1_ [F cm^−2^ s^ϕ−1^]	ϕ_1_	R_ct1_ [Ω cm^2^]	T_2_ [F cm^−2^ s^ϕ−1^]	ϕ_2_	R_ct2_ [Ω cm^2^]	${\bar{C}}_{dl}$ [F cm^−2^]
(2) pH = 5.5	10(1)	5.1(77)·10^−5^	0.79(12)	8.82(92)·10^3^	5.8(87)·10^−5^	0.52(8)	191(29)	7.5·10^−4^
(3) pH = 7.4 + 0.1M NaF	8(1)	4.3(65)·10^−5^	0.74(11)	5.06(76)·10^3^	9.8(97)·10^−5^	0.56(8)	253(38)	3.9·10^−4^
(4) pH = 5.5 + 0.1M NaF	4(1)	2.5(38)·10^−7^	0.66(10)	2.46(37)·10^5^	4.6(69)·10^−5^	0.56(8)	219(33)	1.0·10^−4^
(6) pH = 7.4 + 15 mL Meridol^®^ by Colgate-Palmolive Company, New York, NY, USA	21(3)	8.7(73)·10^−5^	0.83(12)	2.87(43)·10^4^	7.6(94)·10^−5^	0.57(9)	115(17)	2.6·10^−4^

**Table 6:** The key parameters of the corrosion resistance of the Co-Cr-Mo electrode covered with the TiO_2_-ZrO_2_ sol–gel coating sintered at 300 °C in the unmodified and modified saliva solutions at 37 °C, where E_cor_—corrosion potential; j_cor_—corrosion current density; E_p_—protection potential; and E_bd_—breakdown potential (see Figure 10a).

Type of Electrolyte	E_cor_ [V]	j_cor_ [A cm^—2^]	E_p_ [V]	E_bd_ [V]	E_bd_—E_p_ [V]
(1) Saliva pH = 7.4	−0.309(36)	−4.88(58)·10^−8^	0.438(66)	0.797(86)	0.359
(2) Saliva pH = 5.5	0.027(3)	−2.71(33)·10^−7^	0.670(98)	0.799(98)	0.129
(3) Saliva pH = 7.4 + 0.1M NaF	−0.070(8)	−1.50(18)·10^−9^	0.454(68)	0.849(99)	0.395
(4) Saliva pH = 5.5 + 0.1M NaF	−0.292(34)	−3.05(36)·10^−8^	0.545(80)	0.798(95)	0.253
(5) Saliva pH = 7.4 + 15 mL Listerine Total Care Teeth Protection ^®^ by McNeil Consumer Healthcare McNeil-PPC, Inc., Fort Washington, PA, USA	−0.428(52)	−6.71(80)·10^−9^	0.420(65)	0.793(98)	0.373
(6) Saliva pH = 7.4 + 15 mL Meridol^®^ by Colgate-Palmolive Company, New York, NY, USA	−0.482(58)	−1.16(14)·10^−7^	-0.455(78)	0.623(77)	0.623

**Table 7:** The key parameters of the corrosion resistance of the Co-Cr-Mo electrode covered with the TiO_2_-ZrO_2_ sol–gel coating sintered at 500 °C in the modified saliva solutions at 37 °C, where E_cor_—corrosion potential; j_cor_—corrosion current density; E_p_—protection potential; and E_bd_—breakdown potential (see Figure 10b).

Type of Electrolyte	E_cor_ [V]	j_cor_ [A cm^—2^]	E_p_ [V]	E_bd_ [V]	E_bd_—E_p_ [V]
(1) Saliva pH = 7.4	−0.297(39)	−1.17(13)·10^−9^	0.037(6)	0.102(16)	0.065
(2) Saliva pH = 5.5	−0.305(43)	−1.28(14)·10^−9^	−0.111(13)	0.098(14)	0.209
(3) Saliva pH = 7.4 + 0.1M NaF	−0.456(59)	−3.91(43)·10^−7^	0.437(48)	0.794(92)	0.357
(4) Saliva pH = 5.5 + 0.1M NaF	−0.344(44)	−3.51(39)·10^−8^	0.633(72)	0.798(99)	0.165
(5) Saliva pH = 7.4 + 15 mL Listerine Total Care Teeth Protection^®^ by McNeil Consumer Healthcare McNeil-PPC, Inc., Fort Washington, PA, USA	−0.546(73)	−1.95(22)·10^−8^	0.799(88)	0.890(84)	0.091
(6) Saliva pH = 7.4 + 15 mL Meridol^®^ by Colgate-Palmolive Company, New York, NY, USA	−0.406(52)	−1.22(12)·10^−9^	−0.161(19)	0.556(72)	0.717

## Data Availability

The original contributions presented in the study are included in the article, further inquiries can be directed to the corresponding author.
